# Identification and Pathogenicity of Fungal Pathogens Associated with Stem End Rots of Avocado Fruits in Kenya

**DOI:** 10.1155/2020/4063697

**Published:** 2020-07-09

**Authors:** E. K. Wanjiku, J. W. Waceke, B. W. Wanjala, J. N. Mbaka

**Affiliations:** ^1^Department of Agriculture Science and Technology, Kenyatta University (KU), Nairobi, Kenya; ^2^Department of Animal Health and Production, Mount Kenya University (MKU), Nairobi, Kenya; ^3^Biotechnology Research Institute, Kenya Agricultural and Livestock Research Organization (KALRO), Nairobi, Kenya; ^4^Horticulture Research Institute, Kenya Agricultural and Livestock Research Organization (KALRO), Nairobi, Kenya

## Abstract

Losses associated with stem end rot (SER) of avocado fruits have been reported in all avocado growing regions of the world. In Kenya, mature avocado fruits present SER symptoms during storage and marketing, but the disease causal agent(s) has not been established. This study aimed to identify the fungal pathogen(s) associated with avocado SER in Kenya and evaluate its pathogenicity. Fungal isolates were collected from symptomatic avocado fruits from randomly selected orchards and major markets within Murang'a County, a major avocado growing region in Kenya, between September 2017 and March 2018. A total of 207 and 125 fungal isolates, recovered from orchards and major markets, respectively, were identified morphologically and further confirmed by molecular techniques. The identified isolates were *Lasiodiplodia theobromae* (39.8%), *Neofusicoccum parvum* (24.4%), *Nectria pseudotrichia* (18.4%), *Fusarium solani* (7.2%), *F. oxysporum* (5.1%), *F. equiseti* (3.9%), and *Geotricum candidum* (1.2%). *Geotricum candidum* was exclusively recovered from fruits from the market. In the pathogenicity test, *L. theobromae*, *N. parvum,* and *N. pseudotrichia* caused the most severe SER symptoms. Consequently, they were considered to be the major pathogens of SER of avocado fruits in Kenya. To our knowledge, this is the first report of SER pathogen of avocado fruits in Kenya. Given the significant contribution of avocado fruits to household income and foreign exchange in Kenya, this information is significant to further develop management strategies of postharvest loss of avocado fruits in Kenya.

## 1. Introduction

In Kenya, avocado (*Persea americana* Mill.) is one of the most important perennial tropical fruit crops and a major foreign exchange earner. In 2017, it accounted for about 74% by value of the total fruits exported from the country [1]. Currently, “Hass” avocado contributes approximately 80% of the avocado fruits produced and exported from Kenya [2]. Other cultivars produced include “Fuerte,” “Puebla,” “Duke,” and “G6” [3]. Avocado production in Kenya is dominated by smallholder farmers (85%) within several agroecological zones, who mainly produce for the export market, and the remainder is sold in the local markets. Seventy percent (70%) of the avocado fruits are produced in the central and eastern regions of the country. The fruits are exported mainly to the European Union [2, 4]. Since the year 2000, the acreage under avocado production has increased significantly, leading to increased export of the avocado fruit from Kenya [4]. The increased production is fueled by high demand for avocado fruits in the global market due to consumer awareness of the dietary value of the fruit [5]. Despite the increased production and export of avocado fruits from Kenya, high incidences of postharvest fungal diseases, including anthracnose and SER, limit marketing of the fruits and contribute to increased losses by the producers [6, 7].

The symptoms of stem end rot (SER) develop on the avocado fruit as it ripens. It is characterized by shriveling, followed by brown to black rot that starts at the stem end of the fruit. As the rot progresses, internal vascular bundles may have black to brown colorations and eventually the whole fruit is consumed by the rot [8, 9]. Fruits hardly display SER symptoms before harvest. Furthermore, SER often occur at the packing house during transit or after marketing.

Various fungal species have been reported to cause SER on avocado fruits. In Chile, the fungal pathogens reported to cause SER included members of Botryosphaeriaceae family, namely *Diplodia mutila*, *D. pseudoseriata*, *D. seriata*, *Dothiorella iberica*, *Lasiodiplodia theobromae*, *Neofusicoccum australe*, *N*. *nonquaesitum*, and *N*. *parvum* [10]. In Italy, *N*. *parvum*, *Colletotrichum gloeosporioides,* or *C. fructicola* and *Diaporthe foeniculacea* or *D. sterilis* were the most isolated SER pathogens [9]. In California, *Neofusicoccum luteum* and *Phomopsis perseae* were reported [8] while in South Africa, *Thyronectria pseudotrichia*, *Dothiorella aromatica*, *Pestalotiopsis versicolor*, *Lasiodiplodia theobromae*, *Rhizopus stolonifer*, *Fusarium sambucinum*, and *Fusarium solani* were reported [11].

In Kenya, however, the actual pathogen causing SER has not been identified, but on the other hand, anthracnose pathogens have been described [12]. Therefore, this study aimed at identifying the fungal pathogen associated with SER of avocado fruits in the central highlands of Kenya and testing their pathogenicity.

## 2. Materials and Methods

### 2.1. Study Area and Sample Collection

The study was conducted in Murang'a County, which is the leading county in production and export of avocado fruits in Kenya [1]. Geographically, the county lies between latitudes 0°34′ south and 1°07′ south and longitudes 36° east and 37°27′ east, with an elevation of 914 m a.s.l in the east and 3,353 m a.s.l in the west. Avocado fruits are cultivated in the agroecological zones two, three, and four that have 18.0°C to 27.2°C average temperature ranges and 1600 mm–900 mm average annual rainfall [13].

Between September 2017 and March 2018, systematic sampling was used to select 162 orchards included in the study. The orchards had more than five “Hass” avocado fruit trees. Six mature avocado fruits were harvested at random from each five randomly selected avocado fruit trees in every sampled orchard. In addition, 10 “Hass” fruits, at different stages of ripening, were bought from different traders in three major markets (Kandara, Kirwara, and Maragwa) within the county at weekly intervals for two months. A total of 453 fruits from 4,860 fruits harvested from the orchards and 240 fruits from the market were sampled, packed in cartons, and transported to Kenya Agricultural and Livestock Research Organization (KALRO), Kandara, where they were incubated at room temperature (22°C–25°C) for 7–14 days to allow development of SER.

### 2.2. Fungal Isolation

The 207 fruits from the orchards and 125 fruits from the market that displayed SER symptoms were washed with clean tap water, surface-sterilized with 2% sodium hypochlorite for one minute, rinsed in distilled water, and air-dried. Small pieces of flesh from the margins of symptomatic flesh were placed aseptically in 9 cm diameter Petri dishes containing potato dextrose agar (PDA) amended with streptomycin sulfate and incubated at room temperature (22°C–25°C) for five days. Pure cultures were obtained by transferring the mycelia tips on 1.5% (wt/vol) water agar (WA) and allowed to grow overnight. Hyphal tips of the mycelia growth in the WA were later transferred onto PDA amended with streptomycin sulfate. Slant universal bottle was used to preserve the pure cultures of the pathogen and stored in the fridge at 4°C for later use.

#### 2.2.1. Preparation of Conidial Suspension

Fourteen-day-old pure cultures in PDA were flooded with sterile distilled water. A sterile wire loop was used to scrape off the conidia and bring them to suspension. The suspension was filtered through a double-layer muslin cloth and the collected filtrate diluted serially to 1 × 10^5^. A haemocytometer was used to adjust the spore concentration.

### 2.3. Morphological Characterization of the Isolate

To induce conidia production, small pieces of mycelia from the isolates were transferred into 9 cm diameter Petri dishes with PDA amended with autoclaved avocado wood chips and incubated at 25 ± 1°C for four weeks. The isolates were morphologically identified based on cultural and microscopic characteristics as described by Valencia et al. [10], Phillips et al. [14], and Watanabe [15]. Lactophenol blue was used in microscopic identification. The length and width of conidia (*N* = 50) from each isolate were measured using light microscope Zeiss-Primo Star, coupled to AxioCam ERc 5s camera.

### 2.4. Molecular Characteristics

#### 2.4.1. DNA Extraction

An improved fungal extraction protocol described by Innis et al. [16] was used to extract DNA from three representative isolates of each species. Pure fungal cultures derived from the single spores incubated in PDA were used. Forty milligram (mg) of mycelium was placed in a microcentrifuge tube containing 300 *μ*l of extraction buffer (Tris-HCl, 200 mM Ph 8.5; EDTA, 25 mM; 1 M NaCl 250 mM; SDS, 0.5%) with glass beads. The tubes were placed in a fastprep®-24 genogrinder for one minute at 2000 rpm. Two hundred microlitre (*μ*l) of 3 mM sodium acetate pH5.2 was added and refrigerated at −20°C for 10 minutes. After incubation, the samples were centrifuged for 10 minutes at 13000 rpm. After that, the supernatants were transferred into fresh 1.5 ml microcentrifuge tubes. Equal amounts of isopropanol were added to the supernatants and allowed to stand for five minutes at room temperature. After five minutes, the samples were centrifuged for 10 minutes at 13000 rpm and the supernatant was discarded. Five hundred *μ*l of 70% ethanol was then added to the pellets and centrifuged at 13000 rpm for 10 minutes to wash the pellet. The nucleic acid pellets obtained were air-dried and then resuspended in 50 *μ*l of low salt TE buffer (Tris-HCl, 1 mM, pH 8; EDTA, 0.1 mM) and stored at −20°C for later use. The quality of DNA was determined by agarose gel electrophoresis and quantified using a NanoDrop ND-1000Spectrophotometer. DNA was standardized or normalized to 20 ng/*μ*l for polymerase chain reactions (PCR).

#### 2.4.2. DNA Amplification and Sequencing

The extracted DNA was used as templates in PCR. Two sets of primers, ITS1 (*TCCGTAGGTGAACCTGCGG*) and ITS4 (*TCC TCC GCT TAT TGA TAT GC*), ITS5 (*GGA AGT AAA AGT CGT AAC AAG G*) and ITS1, were used in the amplification of the internal transcribed region rDNA of the fungal isolates [16]. PCR reaction volumes of 25 *μ*l containing 2.5 *μ*l of 0.2 *μ*Μ of each primer, 5 × My Taq reaction buffer, 0.25 *μ*l Taq polymerase (Bioline, Meridian Life Science, Memphis, USA), 40 ng/*μ*l of each DNA template, and 12.75 *μ*l of molecular water were used. For amplification, the GeneAmp 9700 DNA Thermal Cycler (Perkin-Elmer) was used. The process involved an initial denaturing step at 94°C for 30 s, followed by 35 cycles, denaturing at 94°C for 30 s, annealing at 55°C for 30 s followed by extension for 1 minute at 68°C, and a final extension step of 5 min at 68°C. To confirm amplification, the PCR products were run on 1.5% agarose gel and visualized under UV light using ENDURO™ GDS. The PCR products were cleaned using the Qiagen PCR cleaning kit according to manufacturer instructions and submitted for Sanger sequencing with forward and reverse primers at Inqaba Africa Genomic platform, South Africa.

#### 2.4.3. Bioinformatics Analysis

Sequence data was analyzed by assigning reads to samples, indexes, primers, and adapters. The primers were marked using Picard (https://broadinstitute.github.io/picard/index.html). Bam2fastq (https://gsl.hudsonalpha.org/information/software/bam2fastq) was used to convert the resultants' bam files to fastq. The overall sequencing quality of the reads was evaluated visually using the Fast QC program (http://www.bioinformatics.babraham.ac.uk/projects/fastqc/). The quality parameters used in filtering the reads included a minimum length of 250 bp and a minimum QC value of 30. Trimming was done corresponding to the adapters and low-quality sequences from all the reads. Subsequent analysis and processing of the reads was done in the CLC Genomic Workbench 11.0, where the overlapping reads were merged. The de novo assembly of the unassembled reads and the raw reads' alignment was performed using CLC Genomics Workbench 11.0 with default parameters (minimum contig = 100 bp, 23 K-mer, similarity fraction = 80%, and length fraction = 50%). BLAST analysis of the ITS sequences was done to support the morphological identification of the samples at the NCBI database. The ITS sequences were deposited in GeneBank using BankIT.

### 2.5. Pathogenicity Test

To establish Koch's postulates, *Geotrichum candidum*, *Fusarium equiseti*, *Fusarium oxysporum*, *Fusarium solani*, *Lasiodiplodia theobromae*, *Neofusicoccum parvum,* and *Nectria pseudotrichia* were subjected to pathogenicity test as described by Freeman et al. [17] and Twizeyimana et al. [8]. Healthy “Hass” fruits were harvested from the farms known to have a low incidence of SER within Murang'a County. The fruits were washed with clean tap water to remove any soil debris. The fruits were surface-sterilized by dipping in 75% ethanol for about three minutes, rinsed with distilled water, and then air-dried. Each of the isolates was subjected to two methods of inoculation.

A sterile cork borer (5 mm diameter) was used to wound the stem end of each fruit and mycelial discs of equivalent diameter obtained from the edge of actively growing pure cultures were placed on the wound. Six inoculated fruits for each pathogen and six control fruits inoculated with plain PDA were arranged on individual trays and covered with cling film to conserve moisture and avoid contamination. The fruits were incubated at room temperature of 24°C ± 1.

**Figure 1:**
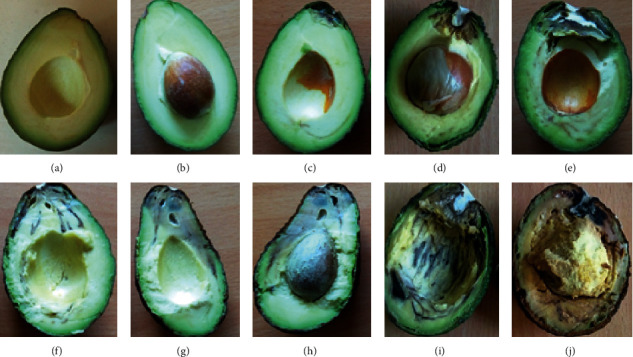
Symptoms of SER on artificial inoculated “Hass” avocado fruits. Guide to severity scoring of the disease rot scale (0–4). (a) and (b) represent 0; (c) and (d) represent 1; (e) and (f) represent 2; (g) and (h) represent 3; (i) and (j) represent 4.

After snapping the pedicel of air-dried fruits, conidial suspension (5 × 10^−5^ conidial/ml) was placed on stem end opening and covered with cling film. Six inoculated fruits for each pathogen and six control fruits inoculated with distilled water were arranged in individual trays and covered with cling film. The inoculated fruits were incubated at 24 ± 1°C. Evaluation was done after 12 days by cutting the fruits longitudinally and rating SER symptoms on a 0–4 rating scale as follows: 0 = no visible rot; 1 = 1–25% rot; 2 = 25–50% rot; 3 = 50–75% rot; 4 = ≥75% rot (Figure 1). At the end of the pathogenicity test, reisolation from the symptomatic fruits was made, and reisolated fungal colonies compared morphologically to the original isolates [8, 9]. SER severity on avocado fruits was calculated using the following formula [18]:

(1) $$\begin{array}{c} \text{percent disease index }\left(\text{PDI}\right)\;=\frac{\text{sum of numerical ratings}}{\text{no. of fruits examined}\times \text{maximum grade}}\times 100. \end{array}$$

### 2.6. Data Analysis

The Sanger sequenced data was analyzed and processed using CLC Genomic Workbench version 11.0. Analysis of Variance (ANOVA) followed by Tukey's post-hoc which was used to compare the mean percentage growth rate of inoculated fungi, while Student's *t*-test was used to compare SER lesions on fruits under different methods of inoculation. Statistical analysis was performed using Min tab v8 (Minitab, LLC).

## 3. Results

### 3.1. Isolation and Identification of Fungal Species

**Figure 2:**
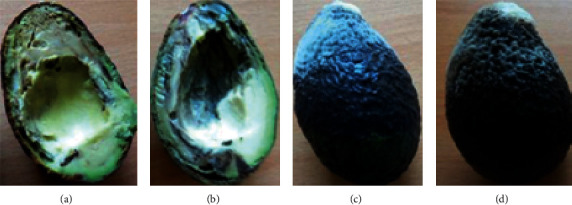
SER symptoms displayed after incubation. (a) brown discoloration of the fruit pulp; (b) black discoloration of the vascular bundles; (c) and (d) fungal mycelia developing on the surface.

After incubation of the fruits for 7–14 days, dark brown to black rot developed on the avocado fruits, and fungal mycelia were occasionally observed on the fruit surface. Internally, a discoloration of the vascular bundles was observed (Figure 2). As the fruit ripened, the rot progressed on the whole fruit. A total of 207 isolates were collected from the fruits from the orchards, and 125 isolates were collected from fruits from the markets.

Based on colony and conidial features the isolates were grouped into seven groups (Table 1).

**Figure 3:**
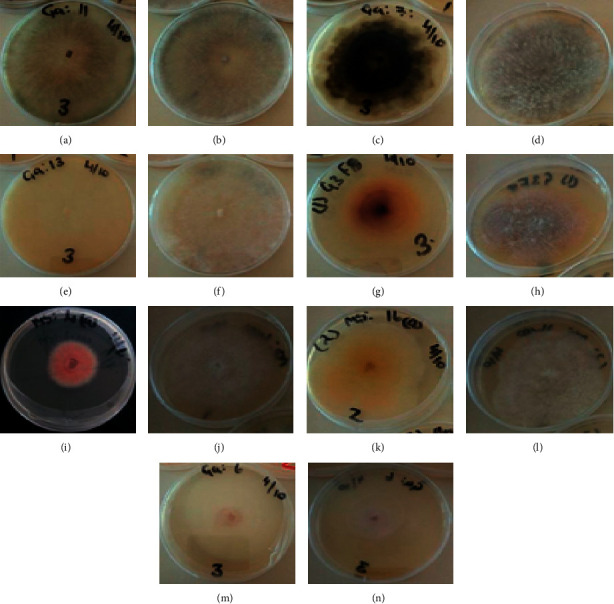
Characteristics of colony (reverse and front) of pathogenic isolates of SER on PDA. (a) and (b) represent reverse and front of *Lasiodiplodia theobromae* (GA11); (c) and (d) *Neofusicoccum parvum* (GA7); (e) and (f) *Nectria pseudotrichia* (GA13); (g) and (h) *Fusarium solani* (1GEF8); (i) and (j) *Fusarium oxysporum* (MS4a); (k) and (l) *Fusarium equiseti* (1GF17); (m) and (n) *Geotricum candidum* (GA6).

Group 1 *Lasiodiplodia theobromae* colony on PDA was round and smooth. At first, white aerial filamentous mycelia with grey center developed. With age, colony turned grey and then dark grey to black (Figures 3(a) and 3(b)). The pycnidia were grey in colour and either simple or aggregated. The conidia were subovoid to ellipsoid. Initially, they were aseptate, thick-walled, and hyaline; however, with time, they formed a single medium septum and became dark brown ranging from 17.35 to 29.31 × 11.23 to 14.91 *μ*m (mean 22.68 × 5.70 *μ*m). The morphological characteristics were consistent with what was described by Valencia et al. [10], Phillips et al. [14], and Watanabe [15].

Group 2 *Neofusicoccum parvum* colony on PDA was rough with irregular margins. Initially, white dense filamentous aerial mycelia developed and turned from dark to black with time (Figures 3(c) and 3(d)). Pycnidia were black, globose, and simple or aggregated. The conidia were bluntly round to subovoid, aseptate, and hyaline with granular content, and with time they turned from light brown to black with a size of 19.77 to 15.25 × 4.10 to 7.5 *μ*m (mean 17.01 × 5.70 *μ*m). The morphological characteristics were consistent with what was described by Valencia et al. [10] and Phillips et al. [14].

Group 3 *Nectria pseudotrichia* colonies on PDA were white, cottony, with filamentous, aerial mycelia growth. The colony growth was regular and rough, with smooth margins (Figures 3(e) and 3(f)). The conidia were ovoid to subovoid with greenish granular content ranging from 6.27 to 12.50 × 2.20 to 9.40 *μ*m (mean 8.49 × 4.95 *μ*m). The morphological characteristics were consistent with what was described by Hirooka et al. [19].

Group 4 *Fusarium solani* colonies on PDA were white, cottony, with floccose mycelium. The colony margins were regular and smooth. The rate of growth was low. The underside was pale to brown in colour (Figures 3(g) and 3(h)). The microconidia were hyaline, oval and some were cylindrical with smooth edges ranging from 5.02 to 8.52 × 2.91 to 5.50 *μ*m (mean 6.88 × 3.79 *μ*m), while the macroconidia were hyaline, slightly curved, and broad with two to three septa reaching within 13.05 to 34.18 × 2.10 to 5.50 *μ*m (mean 18.85 × 3.36 *μ*m). The morphological characteristics were consistent with what was described by Hafizi et al. [20] and Watanabe [15].

Group 5 *Fusarium oxysporum* colonies on PDA were with abundant white to creamy aerial mycelia. The colony margins were smooth and sometimes slightly looped. The reverse side of the colony was pale red to peach violet in colour (Figures 3(i) and 3(j)). Numerous ovoid to kidney-shaped microconidia without septa of 11.2 to 19.9 × 4.5 to 8.4 *μ*m (mean 15.4 × 6.1 *μ*m) were produced. The macroconidia were thin-walled, falcate to almost straight, and both ends were almost pointed with 2-3 septa ranging from 22.1 to 43.9 × 5.1 to 12.5 *μ*m (mean 28.4 × 7.5 *μ*m). The characteristics were similar to what was observed by Hafizi et al. [20], Hussain et al. [21] and Watanabe [15].

Group 6 *Fusarium equiseti* colonies on PDA were white, with abundant cottony mycelium that browned with age. Pale to dark brown diffusible pigmentation was observed (Figures 3(k) and 3(l)). The microconidia were not present; however, long and slender slightly curved at the ends with three to six septa macroconidia of 25.3 to 46.7 × 3.5 to 4.6 *μ*m (mean 37.2 × 3.24 *μ*m) were observed as similarly observed by Motlagh [22] and Watanabe [15].

Group 7 *Geotricum candidum* colonies on PDA were not dense and white to beige appressed onto culture medium with smooth margins (Figures 3(m) and 3(n)). The mycelia formed smooth margined arthroconidia, which were hyaline, one-celled, and subglobose or cylindrical with either rounded or truncated apices reaching 6.1 to 19.7 × 2.3 to 10.3 *μ*m (mean 11.38 × 5.56 *μ*m). The fungus morphological features were consistent with those described by Zhang et al. [23], Alam et al. [24], and Watanabe [15].

Further more, to support the morphological identification of the samples, molecular markers ITS5 and ITS4 and ITS1 and ITS5 were used for molecular identification and consistently yielded high levels of species discrimination. PCR amplification for the ITS yielded products of 526 to 550 bp. From the blast analysis, fungal isolates were able to identify seven species. The isolates reported in this study have been associated with tropical fruits. These included *F. equiseti* (MK922072, MK922069), *F. oxysporum* (MK922065), *F. solani* (MK922070, MK922071, MK922066), *G. candidum* (MK215811, MK922075), *L. theobromae* (MK922068, MK922073), *N. parvum* (MK922067), and *N. pseudotrichia* (MK922074). The closest match between isolates from this study and those mined from the GeneBank had a range from 99 to 100% similarity and are shown in (Table 2).

### 3.2. Pathogenicity Tests

The avocado fruits inoculated with mycelia and those inoculated with spore suspensions developed similar symptoms as observed in fruits obtained from orchards and markets (Figure 1). All the inoculated fruits developed SER symptoms regardless of the isolate or the method of inoculation used. However, disease severity differed across the different fungal species as well as the method of inoculation (Table 3). When inoculated with mycelia, SER severity ranged between 6.67% and 90.83% and when spore suspension was used the severity ranged between 97.50% and 16.67% (Table 3). *Lasiodiplodia theobromae*, *N. parvum,* and *N. pseudotrichia* caused the most severe SER symptoms in both inoculations, and they might be considered the most virulent. No symptoms were observed on the control fruits. Statistical differences (*p* < 0.05) were detected in symptoms development when “Hass” avocado fruits were differentially inoculated with either mycelia or conidial suspensions of *N. parvum*, *N. pseudotrichia*, *F. solani*, *F. equiseti*, *F. oxysporum,* and *G. candidum*. However, there was no statistical significance in symptoms development when inoculated with *L. theobromae* (Table 3).

## 4. Discussion

We report that avocado SER was caused by *Lasiodiplodia theobromae*, *Neofusicoccum parvum*, *Nectria pseudotrichia*, *Fusarium solani*, *Fusarium oxysporum*, *Fusarium equiseti,* and *Geotricum candidum* in the central highlands of Kenya. This is the first report on identification of SER fungal pathogens of avocado fruits in Kenya. The identified pathogens have been associated with SER of avocado fruits in other avocado growing regions in the world such as North America (California), Chile, South Africa, and Italy [8–11]. From the current study, *L. theobromae* was the most frequently isolated pathogen, followed by *N. parvum* and *N. pseudotrichia*. The study corroborates reports by Galsurker et al. [25] that identified *L. theobromae* as an emerging pathogen of fruits SER worldwide. The pathogen has been associated with SER of mangoes and pawpaw [26, 27] and also identified as a major pathogen that causes postharvest disease of many fruits [28].

Further more, results corroborate findings from other avocado growing regions of the world where members of Botryosphaeriaceae family were reported to be the leading cause of SER of avocado fruits. Botryosphaeriaceae species have been reported to cause SER of avocado in South Africa, Italy, California, and New Zealand. In South Africa *N. pseudotrichia* was the most isolated pathogen, and occasionally, *L. theobromae* was isolated. In Italy, California, and New Zealand, *N. parvum* was the most isolated pathogen [8–11, 29]. Temperatures influence SER pathogen predominant in an area. Botryosphaeriaceae species thrive in high temperature, while water stress stimulates latent infections by the species [25, 30]. The avocado fruit production in Murang'a County is concentrated in the lower region of the county, characterized by warm weather and temperature ranges between 18.0°C and 27.2°C [13]. These could explain why *L. theobromae* and *N. parvum* were the most isolated fungal pathogens.

In California *N. parvum* and other species of Botryosphaeriaceae (*N. australe*, *N. luteum*, *Fusicoccum aesculi*, and *Dothiorella iberica*) were associated with SER [8]. However, in our study, only *N. parvum* was isolated, similar to reports on SER pathogen of avocado fruits in Italy [9]. Three *Fusarium* species, namely, *F*. *solani*, *F. oxysporum*, and *F. equiseti*, were found to be minor pathogens of SER in Kenya. Similar findings were reported from South Africa, New Zealand [11, 31], and Ethiopia [30]. *Geotricum candidum* was exclusively isolated from four avocado fruits from the market and never from the orchard fruits. The pathogen has been associated with sour rots of tomatoes, citrus fruits, and vegetables [32]. In the open air markets in Kenya, where the avocado fruits were bought, fruits from avocado, citrus, and other species are placed together, thus allowing for cross-infection between those fruit species.

*Colletotrichum gloeosporioides*, which have been previously reported as causing SER of avocado in Italy and California [8, 11], was not isolated in our study corroborating reports from Chile [10]. Moreover, Twizeyimana et al. [8] identified *C. gloeosporioides* as a weak avocado SER pathogen and is only important when in combination with other SER pathogens.

Morphological characteristics together with DNA analysis were used to identify and differentiate *L. theobromae*, *N. parvum*, and *N. pseudotrichia*. *Lasiodiplodia theobromae* grew fast and colonised the Petri dish in two days. *Neofusicoccum parvum* and *N. pseudotrichia* colonized the Petri dish in four and five days, respectively. The three pathogens showed almost similar morphological features. However, ITS sequences of these fungi clearly allowed the differentiation of the species.

*Lasiodiplodia theobromae* was the most isolated pathogen from fruits from both orchards and markets, followed by *N. parvum* and *N*. *pseudotrichia*. During pathogenicity studies, the three pathogens also caused the most severe SER on avocado fruits. The three pathogens are, therefore, identified as the main causal agents of avocado SER in Kenya.

Further studies should be conducted in other avocado growing regions in the country to get a clear picture of SER etiology in Kenya. Besides, preharvest and postharvest SER management practices of avocado fruits in the country should be established.

## Figures and Tables

**Table 1:** Isolation frequency of SER associated fungi from avocado fruits obtained from orchards and markets.

Group	Isolated fungal pathogen	Orchards		Markets	
		Number of isolates	% isolation	Number of isolates	% isolation
1	*Lasiodiplodia theobromae*	82	39.6	50	40.0
2	*Neofusicoccum parvum*	55	26.6	26	20.8
3	*Nectria pseudotrichia*	39	18.8	22	17.6
4	*Fusarium solani*	14	6.8	10	8.0
5	*F. oxysporum*	10	4.8	7	5.6
6	*F. equiseti*	7	3.4	6	4.8
7	*Geotricum candidum*	0	0	4	3.2
	Total	207	100	125	100

**Table 2:** GenBank accession numbers obtained in this study and those from the NCBI GenBank database used in species identification from ITS sequences.

ITS				
Species	Isolate	This study	GenBank	Percentage similarity
*F. equiseti*	2MS16a	MK922072	MG274307	99
	1GF17	MK922069		
*F. oxysporum*	MS4a	MK922065	MK590412	99
*F. solani*	MS47b	MK922070	GQ229075	99
	MS37a	MK922071	KX688164	99
	1G3F8	MK922066		
*G. candidum*	GA6a	MK215811	HG936031	99
	GA6	MK922075		
*L. theobromae*	GA11	MK922068	KP872340	100
	KA6	MK922073		
*N. parvum*	GA7	MK922067	HQ832811	99
*N. pseudotrichia*	GA13	MK922074	MG800781	99

**Table 3:** Severity of SER on “Hass” avocado based on lesion development on fruit, with different fungal pathogens.

Isolate	Species	SER on avocado fruits	
		Mycelia plugs % SER	Conidial suspension % SER
MK922068	*Lasiodiplodia theobromae*	81.67 ± 4.4^Aa^	84.17 ± 9.70^ABa^
MK922067	*Neofusicoccum parvum*	80.83 ± 2.39^Ab^	97.50 ± 1.12^Aa^
MK922074	*Nectria pseudotrichia*	90.83 ± 4.55^Aa^	74.17 ± 5.39^Bb^
MK922066	*Fusarium solani*	28.33 ± 3.80^Bb^	60.80 ± 11.2^Ca^
MK922065	*Fusarium oxysporum*	5.00 ± 0.00^Ca^	19.2 ± 6.7^Eb^
MK922069	*Fusarium equiseti*	24.17 ± 0.83^Bb^	41.67 ± 6.91^Da^
MK922075	*Geotricum candidum*	6.67 ± 1.05^Cb^	16.67 ± 3.07^Ea^
Control	Control	0.00 ± 0.00^C^	0.00 ± 0.00^F^

Values were expressed as mean ± standard error of mean. Values followed by different lowercase superscripts across the rows and different uppercase superscripts along the columns are significantly different at *p* < 0.05.

## Data Availability

The data used to support the findings of this study are included in the article.
